# Periprosthetic Joint Infection Caused by Trichoderma: Is It Plausible?

**DOI:** 10.3390/diagnostics15202575

**Published:** 2025-10-13

**Authors:** Iveta Tiepelyte, Tomas Kadusauskas, Aurimas Sirka, Rihard Trebse, Jaime Esteban, Gintare Sinkute, Danguole Vaznaisiene

**Affiliations:** 1 Department of Infectious Diseases, Lithuanian University of Health Sciences, LT-47116 Kaunas, Lithuania; 2 Department of Health Promotion and Rehabilitation, Lithuanian Sports University, LT-44221 Kaunas, Lithuania; 3 Department of Orthopedics and Traumatology, Kaunas Hospital of the Lithuanian University of Health Sciences, LT-47114 Kaunas, Lithuania; 4 Department of Orthopaedics and Traumatology, Lithuanian University of Health Sciences, LT-50161 Kaunas, Lithuania; 5 Orthopaedic Hospital Valdoltra, SI-6280 Ankaran, Slovenia; 6 Faculty of Medicine, University of Ljubljana, SI-1000 Ljubljana, Slovenia; 7 Department of Clinical Microbiology, IIS-Fundación Jiménez Díaz, CIBER de Enfermedades Infecciosas (CIBERINFEC), ES-28040 Madrid, Spain; jestebanmoreno@gmail.com; 8 Department of Laboratory Medicine, Lithuanian University of Health Sciences, LT-50161 Kaunas, Lithuania

**Keywords:** *Trichoderma*, PJI, fungal infection, diagnostics

## Abstract

**Background and Clinical Significance:** *Trichoderma* is a rare cause of human fungal infections. These infections are often severe and can be life-threatening, involving various organs and tissues. Although *Trichoderma* is not commonly encountered in clinical practice, its ability to cause infections is particularly significant in immunocompromised patients. As a result, cases in which *Trichoderma* is identified in human specimens are not only uncommon but also pose complex diagnostic and therapeutic challenges for clinicians. Although it can be associated with various organs, its clinical significance in joints, especially in periprosthetic joint infections, remains unclear. **Case Presentation:** This case report presents a 63-year-old immunocompromised woman with a suspected periprosthetic joint infection caused by *Trichoderma*. After total hip arthroplasty and subsequent bacterial periprosthetic joint infection, *Trichoderma* was revealed from two intraoperative samples. Given the unusual pathogen and the patient’s immunosuppression, antifungal therapy with amphotericin B was initiated. However, due to severe intolerance, the antifungal treatment had to be discontinued shortly after initiation, and the patient continued under close monitoring. We closely monitored the patient’s clinical status and laboratory test results, continuously considering whether the findings represented contamination or true infection. The patient exhibited clinical and radiological stability, reinforcing the hypothesis of contamination rather than active infection. **Conclusions:** This case explores the rarity of *Trichoderma* periprosthetic joint infections, the diagnostic and treatment challenges, and the importance of multidisciplinary input in managing such uncommon and complex cases. Further studies are needed to clarify the pathogenic significance, optimal management, and long-term outcomes of *Trichoderma* in bone and joint infections.

## 1. Introduction

*Trichoderma* is an environmental fungus rarely implicated in human infections. It is primarily known for causing infections in immunocompromised hosts, such as those undergoing organ transplantation or chemotherapy. Although *Trichoderma* infections have been reported in the lungs, sinuses, brain, and other organs, its role as a pathogen in joint infections, especially periprosthetic joint infections (PJI), remains undocumented until now [1,2,3].

PJI is a serious complication following arthroplasty. The most common pathogens are *Staphylococcus aureus* and coagulase-negative staphylococci, but less frequent microorganisms may also be involved. Some of these organisms, which in other clinical settings are usually regarded as contaminants, can act as true pathogens in PJI. Many causative bacteria are able to form biofilm on prosthetic surfaces, thereby complicating both diagnosis and treatment. In clinical practice, careful distinction between contamination and infection is crucial, especially when uncommon and often low-virulence microorganisms are isolated.

In this context, the isolation of *Trichoderma* raises a critical diagnostic dilemma: does it represent more contamination or an active infection warranting antifungal treatment. This distinction is essential, as treatment protocols are not standardized and prolonged antifungal therapy may carry significant toxicity. Amphotericin B is generally considered the first-line agent due to its antifungal activity; nevertheless, alternative antifungal agents are often employed and have been associated with satisfactory clinical results [4,5].

This case presents the first reported suspicion of a PJI caused by *Trichoderma*, which raises the question of whether the fungal growth represents contamination or true infection. Given the lack of literature on *Trichoderma* in such infections, this case underscores the need for multidisciplinary discussions and ongoing surveillance in managing complex fungal infections in immunocompromised patients. The case report is structured according to the CARE guidelines. Documenting such rare cases is essential for guiding future diagnostic approaches, informing antifungal stewardship, and improving recognition of uncommon pathogens in immunocompromised hosts.

## 2. Case Report

Initial patient data. A 63-year-old woman with a complex medical history, including rheumatoid arthritis treated with long-term glucocorticoid therapy, underwent a left total hip arthroplasty in April 2023. Her immunocompromised status, compounded by chronic comorbidities such as diabetes mellitus and chronic kidney disease, placed her at a heightened risk for postoperative infectious complications.

Approximately six months after the initial surgery, in October 2023, the patient presented with symptoms indicative of a PJI. The joint aspiration culture revealed *Staphylococcus epidermidis* resistant to methicillin, which was subsequently identified in four out of six intraoperative samples.

**Figure 1:**
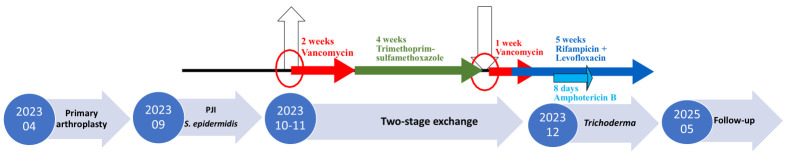
The course of the disease.

Initial treatment involved a two-stage revision surgery. After the first stage, vancomycin was administered, followed by trimethoprim-sulfamethoxazole. A revision bone cement containing 0.5 g gentamicin and 2 g vancomycin was used during the procedure. The second-stage reimplantation surgery took place in November 2023. Vancomycin was continued initially, then switched to a combination of rifampicin and levofloxacin (Figure 1).

Diagnosis. Unexpectedly, three weeks after the second-stage surgery, microbiological cultures from two out of five intraoperative samples revealed the presence of *Trichoderma*, a rare fungus not commonly associated with PJI. Trichoderma was identified by microscopy. Given the patient’s immunocompromised status, this finding raised significant concern regarding a possible fungal infection, which could complicate treatment and prognosis. Consequently, an international multidisciplinary consultation involving infectious disease specialists, microbiologists, and orthopedic surgeons was sought to formulate an optimal management plan.

Treatment. In response to the fungal identification, antifungal therapy with amphotericin B was initiated due to its broad-spectrum activity and effectiveness against rare fungi. However, the patient developed severe intolerance to amphotericin B that necessitated discontinuation of the drug after only eight days of treatment. Despite this setback, the patient continued antibacterial therapy with rifampicin and levofloxacin, aiming to control the bacterial infection.

Outcome. After eighteen months follow-up after the reimplantation procedure, the patient remained asymptomatic, with no clinical or laboratory signs of infection recurrence. Inflammatory markers and radiological imaging showed no evidence of ongoing infection. Continued monitoring was required to determine whether the *Trichoderma* growth represented true infection or contamination.

## 3. Discussion

*Trichoderma* is an opportunistic fungus with a well-documented ability to cause serious infections in immunocompromised patients. Although most cases occur in these patients, some have been described in immunocompetent individuals [1]. Previous case reports have documented fatal outcomes from *Trichoderma* infections in the lungs, brain, and other organs, particularly in transplant recipients. However, its role in causing joint infections, especially in the context of PJI, is unprecedented. A few cases of implant associated infections have been described, but no cases of PJI have been reported.

The diagnosis of *Trichoderma* infection in this case was challenging. The presence of *Trichoderma* in intraoperative samples raised concerns about whether it was a true pathogen or a contaminant, as environmental fungi can easily contaminate cultures. Nevertheless, the decision to initiate antifungal therapy was based on the patient’s immunocompromised status, the potential severity of fungal infections in such hosts and regarding the growth of the pathogen in two intraoperative cultures. This meets the confirmed infection criterion according to the European Bone and Joint Infection Society (EBJIS) definition of periprosthetic joint infection [6]. The case highlights a key diagnostic challenge—discerning between contamination and true infection. The presence of *Trichoderma* in two samples and the patient’s immunocompromised state warranted antifungal therapy initiation to prevent potential progression, although fungal PJIs often have a more insidious or indolent course. However, the patient’s stable condition following early discontinued treatment and lack of recurrent symptoms at the eighteen-month follow-up could suggest that *Trichoderma* contamination, rather than true infection, occurred. In general, there are no approved clinical breakpoints for antifungal susceptibility testing for *Trichoderma*. Diagnosis and identification of *Trichoderma* species as an etiological agent may occur late. In our presented case, the pathogen revealed only after three weeks.

The patient did not show any clinical signs of onychomycosis, which can act as a reservoir for *Trichoderma*. As in all surgical procedures, multiple cultures were obtained to differentiate infection from contamination. Contact with laboratory staff was limited to the routine inoculation of the specimens onto culture media. In addition, this pathogen had not been identified in our laboratory in recent times. We did not have sufficient data to confirm contamination.

Based on recently published reviews, *Trichoderma* infections have been reported in a variety of organs, including the liver, brain, sinuses, ears, lungs, kidneys, spleen, pretracheal region, gastrointestinal tract, skin, eyes, oral cavity, heart (endocardium and pericardium), peritoneum, mediastinum, central nervous system, and lower limbs, fungaemia and disseminated cases have also been reported [1,2,3]. There are data that *Trichoderma* was revealed in two bone samples (sternum, vertebral body), but no clinical data, significance or further course are described [5,7].

A review of published cases shows that *Trichoderma* infections involving implanted medical devices are exceptionally rare but clinically significant. The cases were mainly associated with cardiac implants. Reported implant-associated infections include prosthetic valve endocarditis (with valve culture positive), implantable cardioverter-defibrillator endocarditis (tips of the leads positive by sonication), aortic graft and pacemaker infections. A case involving a cerebrospinal fluid shunt device has also been reported. In these cases, positive microbiological evidence was consistently obtained from device-related samples, confirming true infection rather than contamination. Immunosuppression status was not specified for some patients; in certain cases, information on the exact antifungal regimen and its duration was unavailable [8,9,10,11] (Table 1). The number of positive cultures is often not reported. It should be noted that in one reported case, an additional pathogen *Staphylococcus epidermidis* was identified both from blood and the implant samples (standard and sonication), whereas *Trichoderma* was isolated only from implant sonication fluid, making it difficult to assess the exact role of *Trichoderma* as the causative agent [8]. Bacteria generally grow better than fungi as biofilms. Nevertheless, *Trichoderma* produces biofilms on different surfaces, including implanted medical devices, catheters, aortic conduits, pacemaker sacs, and soft contact lenses. In these cases, sonication from these surfaces is required for release and growth of *Trichoderma* in culture [8]. Sonication of removed implants is useful to dislodge biofilms and improve detection of microorganisms when infection is suspected; however, in some settings it may not be feasible. Twenty co-infections with *Trichoderma* were described with bacteria, fungi, virus, bacteria and virus, fungi and virus, and with bacteria, fungi and virus [5]. The following bacteria were mentioned: *Staphylococcus*, *Escherichia coli*, *Pseudomonas*, *Acinetobacter*, *Klebsiella*, *Clostridium*, *Enterococcus*. The general prognosis for co-infections was poor [5].

In contrast, a larger proportion of reported *Trichoderma* device-related infections involve indwelling catheters and extracorporeal circulation devices, such as peritoneal dialysis catheters, tunneled hemodialysis catheters, ECMO (extracorporeal membrane oxygenation) cannulas, port-a-caths and other catheters [1,2]. These infections generally presented with positive cultures from blood or dialysate fluid, and management uniformly required device removal alongside antifungal therapy. While many of these patients recovered, fatalities occurred, particularly in those with severe underlying disease or delayed diagnosis.

The therapeutic approach to *Trichoderma* infections generally includes amphotericin B as the first-line treatment, with other antifungals such as itraconazole, voriconazole, or caspofungin used as alternative options [4,5]. Amphotericin B remains the primary treatment, although its nephrotoxicity and infusion-related side effects can limit its use. In this case, the patient’s intolerance to amphotericin B forced discontinuation, leaving her on antibacterial therapy alone. This posed a significant clinical dilemma, as untreated fungal infections can lead to increased risk of complications and mortality in immunocompromised patients, on the other hand, the possibility of contamination was not ruled out. Liposomal formulations of amphotericin B are preferred in many cases due to their reduced nephrotoxicity, particularly in patients who cannot tolerate the standard formulation. Surgical intervention is frequently required for abscesses and deep infections, implant removal for implant-associated infections.

Despite aggressive antifungal therapy, the prognosis remains poor in many cases, particularly when *Trichoderma* causes disseminated or necrotizing infections. Nevertheless, successful outcomes are possible with early diagnosis, combination therapy, and surgery where appropriate.

This case adds to the growing evidence that *Trichoderma* can cause invasive infections in vulnerable hosts, though its role in bone and joint, and PJI is still uncertain.

The clinical case had some shortcomings. A histological examination was not performed, which could have provided additional value for the diagnosis, although interpretation of the findings would not be easy due to the concomitant treatment of *S. epidermidis* PJI. Another limitation was that the intraoperative samples were possibly taken with the same surgical instrument, but growth occurred in two of the five specimens, even though the others were collected in the same way. In the reported cases in the literature, information on the number of positive samples was often not provided; in one case, growth was observed only from sonication. Another limitation was that antimicrobial susceptibility could not be determined. In addition, the absence of a molecular method for the identification of *Trichoderma* was also a limitation of this case. Finally, at that time, there was no possibility of switching the treatment to liposomal amphotericin B or another antifungal drug in the hospital, but the possibility of contamination remained, so the treatment was stopped. In the reported cases in the literature, there were instances where voriconazole was insufficiently effective and was switched to amphotericin B, which was subsequently discontinued due to intolerance. Additionally, extending the treatment for a sufficient duration would not help determine whether it was an infection or contamination as in the reported case in the literature [8]. The relative paucity of virulence of *Trichoderma* spp. in immunocompetent hosts is suggested after the report of an inadvertent infusion of *T*. *viride* in a contaminated intravenous solution. This patient received a single dose of amphotericin B and remained well [4,12].

## 4. Conclusions

This case represents the first documented instance of a suspected *Trichoderma* PJI, highlighting the rarity and clinical significance of such fungal involvement in prosthetic joint complications. The unexpected identification of *Trichoderma* in intraoperative cultures raised substantial concerns among the treating team, given the organism’s uncommon nature as a human pathogen and the patient’s immunocompromised status, which typically predisposes to opportunistic infections. Despite these concerns, the patient’s stable clinical condition following the discontinuation of antifungal therapy suggests two possible scenarios: either the *Trichoderma* growth was a result of contamination during sample collection or processing, or the patient’s immune system was sufficiently robust to contain and control the fungal presence, and surgical debridement together with short-term antifungal therapy likely contributed to the resolution of the infection. This case is limited by the absence of histopathological confirmation and the lack of a susceptibility profile for the microorganism. It underscores the value of an international multidisciplinary approach encompassing orthopaedic surgery, infectious diseases, and microbiology in the management of rare implant-associated infections. Collaboration among these specialties not only facilitates timely diagnosis and tailored treatment but also contributes to the growing body of knowledge needed to better characterize the clinical significance and natural history of underrecognized fungal pathogens in PJI. Further research is required to better understand the role of *Trichoderma* in bone and joint infections, and the best treatment strategies for managing such rare and challenging cases in immunocompromised patients.

## Figures and Tables

**Table 1:** Reported cases of *Trichoderma* implant-associated infections in the literature.

Clinical Case Report (Author, Year)	Patient Sex and Age	Immuno- Suppression	Microorganism, Samples Positive	Infected Organ/Device	Other Concomitant Pathogens, Samples Positive	Antifungal Treatment	Surgery	Outcome
Tascsini C 2016 [8]	Male, 30 years old	No	*Trichoderma longibrachiatum*; Tips of the leads (only sonication)	Implantable cardioverter-defibrillator–endocarditis	*Staphylococcus epidermidis*; Tips of the leads (standard and sonication) and blood	Oral voriconazole 200 mg bid (after 400 mg bid loading, day 1), 3 d → 200 mg bid IV, 2 d—both stopped due to subtherapeutic levels; switched to liposomal amphotericin B 3 mg/kg/day IV, 25 d, stopped for renal insufficiency	Implantable cardioverter-defibrillator removal and reimplantation (day 10)	Recovered
Bustamante-Labarta MH 2000 [9]	Male, 66 years old	No	*Trichoderma* spp.; Removed material; Number of cultures NA; Blood cultures negative	Aortic graft-endocarditis	No	Antifungal drugs in the postoperative period; Antifungal and duration: NA	The graft was replaced	Recovered
Hatvani L 2019 [10]	Male, 71 years old	Not reported	*Trichoderma bissettii*; Removed aortic valve; Number of cultures NA; Blood cultures negative for fungi	Prosthetic aortic valve endocarditis	Not reported	Voriconazole IV; Duration: NA	Aortic valve removal	Died due to another cause
Hatvani L 2019 [10]	Female, 75 years old	Not reported	*Trichoderma bissettii*; Fluid from the implant; Number of cultures NA	Pacemaker	Not reported	NA	The device was extracted	NA
Piens MA 2004 [11]	Male, 61 years old	No	*Trichoderma reesei*; Cerebrospinal fluid and shunt device culture	Cerebrospinal fluid shunt device	Not reported	Two-months regimen with 3 antifungal drugs	Cysternalventriculostomy	Recovered

## Data Availability

Dataset available on request from the authors.
